# Are there national strategies, plans and guidelines for the treatment of hepatitis C in people who inject drugs? A survey of 33 European countries

**DOI:** 10.1186/1471-2334-14-S6-S14

**Published:** 2014-09-19

**Authors:** Mojca Maticic, Jerneja Videcnik Zorman, Sergeja Gregorcic, Eberhard Schatz, Jeffrey V Lazarus

**Affiliations:** 1 Clinic for Infectious Diseases and Febrile Illnesses, University Medical Centre Ljubljana, Ljubljana, Slovenia; 2 Correlation Network, Foundation De Regenbook Groep, Amsterdam, The Netherlands; 3 CHIP, Centre for Health and Infectious Disease Research and WHO Collaborating Centre on HIV and Viral Hepatitis, Rigshospitalet, University of Copenhagen, Copenhagen, Denmark

## Abstract

**Background:**

Hepatitis C virus (HCV) infection represents a major global health problem, which in high-income countries now mostly affects people who inject drugs (PWID). Many studies show that the treatment of HCV infection is as successful among PWID as among other populations and recently PWID have been included in the international guidelines for the treatment of HCV infection. The aim of this survey was to collect data from European countries on the existence of national strategies, action plans and clinical guidelines for HCV treatment in the general population and PWID in particular.

**Methods:**

Thirty-three European countries were invited to participate. Data on available national strategies, action plans and guidelines for HCV treatment in general population and in PWID specifically were collected prospectively by means of a structured electronic questionnaire and analyzed accordingly.

**Results:**

All of the 33 invited European countries participated in the survey. Twenty-two responses came from non-governmental organizations, six from public health institutions, four from university institutions and one was an independent consultant. Fourteen (42.4%) of the countries reported having a national strategy and/or national action plan for HCV treatment, from which ten of them also reported having a national strategy and/or national action plan for treatment of HCV infection in PWID. Nearly three-quarters reported having national HCV treatment guidelines. PWID were included in the majority (66.7%) of the guidelines. Fourteen (42.4%) countries reported having separate guidelines for the treatment of HCV infection in PWID.

**Conclusions:**

Given the high burden of HCV-related morbidity and mortality in PWID in Europe, the management of HCV infection should become a healthcare priority in all European countries, starting with developing or using already-existing national strategies, action plans and guidelines for this population.

## Introduction

Hepatitis C virus (HCV) imposes a considerable disease burden worldwide and is one of the major underlying causes of chronic liver disease-related death [1]. Approximately 50-80% of individuals exposed to HCV develop chronic infection, and cirrhosis occurs in 10-15% of these cases within the next 20 years [2,3]. The rate of progression to cirrhosis is highly variable and is influenced by several factors [3]. Persons with cirrhosis are at increased risk for developing end-stage liver disease and hepatocellular carcinoma, the sixth most common cause of cancer globally [3,4]. Chronic HCV infection remains the leading cause of liver transplantation; in 2004, one-quarter of liver transplants performed in 25 European countries were attributable to HCV [5,6].

It has been estimated that between 130 and 170 million people are chronically infected with HCV worldwide and that 350,000 deaths occur each year as a result of HCV [7]. A 2009 analysis of data from 22 European countries concluded that as many as nine million people in the focal area may be HCV-infected [4]. The World Health Organisation (WHO) HCV prevalence estimates for 32 European countries indicated higher HCV prevalence in southern and eastern Europe than in northern Europe [8]. HCV was thought to cause an estimated 86,000 deaths annually in the entire European region [9].

After it became routine to screen blood products for HCV in developed countries, intravenous drug use replaced blood transfusion as the main HCV transmission route in these countries [9,10]. Injecting drug use is a major public health problem around the world as almost 16 million people injected drugs in 2007 [11]. According to 2010 estimates, five million European people who inject drugs (PWID) are HCV antibody-positive, with annual incidence varying from 5% to 45% [11,12]. With 50% to 80% of PWID infected in the developed world, HCV is much more prevalent than either hepatitis B virus infection or HIV in this high-risk population. Furthermore, HCV sero-prevalence is much higher in PWID than in the general population [13,14].

Research indicates that HCV treatment outcomes for PWID are comparable to those for other patients in terms of adherence to treatment, completion of the full treatment course and attainment of a sustained viral response (SVR) [15,16]. Moreover, modelling studies suggest that treatment of active PWID could control the burden of HCV in this population by decreasing the viral load among current drug injectors and reducing transmission rates [17]. Therefore, PWID should represent one of the most important target populations for treatment. Since the cohorts of non-treated PWID with chronic hepatitis C are ageing they represent a significant proportion of patients with advanced liver disease and liver-related mortality [18,19]. Twenty to 25% of deaths among PWID are related to the latter [20]. Further, daily use of cannabis may be associated with advanced liver fibrosis, and heavy alcohol consumption which is commonly present in PWID is associated with a higher risk of cirrhosis [21,22]. However, many clinicians are reluctant to treat hepatitis C in PWID because of concerns regarding re-infection, serious side-effects, potential lack of adherence to treatment, high rates of concomitant alcohol abuse and mental health issues [23].

National HCV treatment strategies, action plans and clinical guidelines that recognise PWID as a distinct patient population can help foster an enabling environment in which PWID are more likely to be given equal access to treatment [24]. Little is known about the extent to which these tools and resources are in place in European countries. The aim of our study was to collect data from European countries on the existence of national HCV treatment strategies, action plans and clinical guidelines for both the general population and PWID.

## Methods

The study was performed between September and December 2013. Data were collected by means of a structured electronic questionnaire that was sent via e-mail to individuals in 33 European countries. In the survey Scotland was treated separately from the United Kingdom and was presented as one of the participating countries due to its well-recognised autonomous system for the management of hepatitis C that is covered by the Scottish National Healthcare System. The data from the United Kingdom excluded the data for Scotland. Informants were drawn from a database of contacts maintained by the Hepatitis C Initiative of the Correlation Network, and were selected because their work deeply involves HCV/PWID issues.

The questionnaire was prepared at the Clinic for Infectious Diseases and Febrile Illnesses, University Medical Centre Ljubljana, Slovenia. It was constructed as a nine-item survey in which answers could only be “yes” or “no” (Table 1). Respondents were asked to provide references for national guidelines on HCV treatment and had the option of adding comments to clarify their answers. The survey also requested the names, organisational affiliations and countries of respondents. The questionnaire was written in English since no language barriers were expected from survey recipients.

**Table 1:** Survey questions

Does your country have a national strategy for treatment of HCV?	yes/no
If yes, does it include actions with regards to PWID?	yes/no
Does your country have a national action plan for treatment of HCV (in place of or in addition to a strategy)?	yes/no
If yes, does it include actions in regard of PWID?	yes/no
Does your country have national guidelines for treatment of HCV?	yes/no
If yes, does this include guidelines in regard of PWID or are these separate guidelines in regard of PWID:	
Guidelines in regard of PWID are included	yes/no
Separate guidelines in regard of PWID	yes/no
In case there are separate guidelines in regard of PWID, are they applicable to:	
PWID on opiate substitution treatment	yes/no
Active PWID	yes/no

HCV: hepatitis C virus. PWID: people who inject drugs.

PWID were defined as either people who were “actively” injecting drugs, referring to those who had used drugs in the past six months, or as people who were former injectors, including those who were still active non-injection drug users and/or used opioid substitution treatment (OST). The definition was taken from recent recommendations for the management of HCV infection in PWID published by the International Network on Hepatitis in Substance Users (INHSU) [25].

Data were collected by the Hepatitis C Initiative of the Correlation Network and then were reviewed and analysed by the authors of the questionnaire.

## Results

Survey responses were obtained from all targeted countries. One survey was received from each of 31 countries. For two other countries, Finland and Italy, two people submitted surveys. One respondent from Finland represented a university-based institution and the other represented a public health institution; both respondents from Italy represented nongovernmental organisations (NGOs). The two Finnish respondents provided the same answers, as did the two Italian respondents. Each set of identical submissions was combined into a single record for Finland and a single record for Italy, with the two respondents regarded as a single respondent. The Finland submission counted as a university institution since more detailed comments were provided by the respondent from there. Hence, the final dataset contained one record and one respondent per country for 33 countries.

Respondents reported various affiliations. About two-thirds of them (22; 66.7%) represented NGOs. Six (18.2%) were based at public health institutions, four (12.1%) were based at university institutions, and one (3%) functioned as a freelance trainer and consultant.

The results of the survey are summarized in Table 2.

**Table 2:** Survey results

Country*	National strategy for HCV treatment / PWID included	National action plan for HCV treatment / PWID included	National HCV treatment guidelines	National HCV treatment guidelines: PWID included	Separate national HCV treatment guidelines for PWID	Separate PWID guidelines are applicable to PWID on OST	Separate PWID guidelines are applicable to active PWID
**Albania** *NGO*	N/N	Y/N	Y	Y	N	Y	N

**Austria** *NGO*	Y/Y	na/na	Y	na	na	na	na

**Belgium **[26] *NGO*	N/N	N/N	N	N	na	na	na

**Bosnia and Herzegovina **[27] *University*	Y/Y	Y/Y	Y	Y	N	na	na

**Bulgaria** *Public health*	N/na	Y/N	Y	na	Y	na	na

**Croatia** *NGO*	N/na	N/na	Y	Y	na	na	na

**Czech Republic **[28-30] *University*	N/N	N/N	Y	N	Y	Y	Y

**Denmark **[31,32] *Public health*	Y/Y	Y/Y	Y	Y	Y	na	Y

**Estonia **[33] *NGO*	N/N	N/N	N	N	N	N	N

**Finland **[34] *NGO*	N/N	N/N	N	N	N	N	N

**France **[35,36] *NGO*	Y/Y	Y/Y	Y	Y	N	na	na

**Germany **[37,38] *NGO*	N/N	N/N	Y	Y	N	na	na

**Greece **[39] *Public health*	Y/Y	Y/Y	Y	Y	Y	Y	N

**Hungary **[40] *NGO*	N/N	N/N	Y	Y	Y	Y	Y

**Ireland **[41] *NGO*	Y/Y	Y/N	Y	N	Y	Y	na

**Italy **[42,43] *NGO*	N/N	N/N	N	N	N	N	N

**Latvia **[44] *NGO*	Y/N	N/N	Y	Y	Y	Y	N

**Lithuania **[45] *University*	Y/Y	N/N	Y	Y	Y	Y	Y

**Macedonia** *NGO*	N/N	N/na	Y	Y	Y	Y	Y

**Montenegro** *NGO*	N/N	N/N	N	N	N	N	N

**Norway** *NGO*	Y/Y	N/N	Y	Y	Y	na	Y

**Poland **[46] *NGO*	N/N	N/N	Y	Y	N	Y	na

**Portugal** *NGO*	N/N	N/N	Y	Y	N	na	na

**Romania **[47] *NGO*	N/N	N/N	N	na	na	Y	na

**Scotland **[48] *Public health*	Y/Y	Y/Y	Y	Y	Y	Y	Y

**Serbia** *NGO*	N/N	N/N	N	Y	na	na	Y

**Slovakia **[49] *NGO*	N/N	N/N	Y	Y	Y	na	na

**Slovenia **[50,51] *University*	Y/Y	Y/Y	Y	Y	Y	Y	Y

**Spain **[52] *Public health*	N/N	N/N	Y	Y	N	Y	N

**Sweden** *NGO*	N/N	N/N	N	N	N	N	N

**Switzerland** *Public health*	N/N	N/N	N	Y	na	Y	na

**The Netherlands **[53-55] *NGO*	N/N	N/N	Y	Y	Y	Y	na

**United Kingdom **[56-58] *Freelance trainer/consultant*	Y/Y	Y/Y	Y	Y	N	na	na

*If respondents provided references to national guidelines, the citations are given in brackets following country names. All respondent affiliations are indicated in italics following country names.

N: no. Y: yes. na: no answer. NGO: non-governmental organization. HCV: hepatitis C virus. PWID: people who inject drugs. OST: opioid substitution treatment.

### National strategy for treatment of HCV infection

Twelve countries (36.4%) reported having a national HCV treatment strategy. All but one of them reported that PWID are included in the strategy. The remaining 21 countries (63.6%) reported not having a national HCV treatment strategy.

### National action plan for treatment of HCV infection

Ten countries (30.3%) reported having a national action plan for HCV treatment. In seven of the ten countries, this plan was reported to include PWID. Respondents from two additional countries, Croatia and Finland, indicated that their countries were in the process of developing a national strategy and action plan. The respondent from Italy stated that his country was currently waiting for the government to approve a national strategy and action plan. The respondent from Austria did not answer this question.

### National guidelines for treatment of HCV infection

Twenty-four countries (72.7%) were said to have national guidelines for treatment of HCV infection. Among the nine countries that reported not having national HCV treatment guidelines, some respondents commented on guidance that was in the process of being prepared. According to the respondent from Romania, a non-governmental document that is well-recognised and appreciated among clinicians provides guidance on treatment decisions in that country. The respondent from Finland reported that country’s national guidelines to be under development. In Italy, national guidelines reportedly had been prepared but had not yet been approved by the government. Until approval is granted, clinicians are expected to follow regional guidelines since the national health service in Italy is based on regional laws. In Switzerland, an awareness campaign on HCV treatment exists for professionals on a national level; further, an analyses of the situation regarding prevention and treatment of HCV were being carried out by the federal authorities, with the purpose of creating guidelines for HCV treatment in the general population and PWID based on the results of the analyses.

### National guidelines for treatment of HCV infection in people who inject drugs

Among the 24 countries reported to have national guidelines for HCV treatment, 20 countries were said to address HCV treatment for PWID in their guidelines. Two additional countries, Serbia and Switzerland, had contradictory information. In both cases, respondents indicated that national guidelines for HCV treatment did not exist, but that PWID were included in the guidelines. Fourteen of the 33 countries (42.4%) were reported to have separate guidelines for the treatment of HCV in PWID; among them, 11 reported including PWID in the national guidelines for HCV treatment.

A survey question asking if there are separate guidelines for PWID who are on opiate substitution treatment (OST) received 15 “yes” responses (45.5% of countries). Five other countries had responses of “no,” and the question was not answered by respondents from the remaining countries. Nine countries (27.3%) were reported to have separate guidelines for treatment of HCV infection in active drug users. Among the remaining countries, respondents from nine countries indicated that there were not separate guidelines; no answer was provided in the other cases.

## Discussion

During the last two decades, it appears that low HCV treatment uptake among PWID in developed countries has contributed to high levels of advanced liver disease and liver disease-related mortality, with 20-25% of deaths in this population attributable to liver disease [59-61]. In general, not accessing HCV treatment has been associated with several barriers arising mostly from poor knowledge and inaccurate perceptions about HCV [62].

Seven respondents involved in this survey (Denmark, France, Greece, Ireland, Scotland, Slovenia and the United Kingdom excluding Scotland) reported having both a national strategy and a national action plan for treatment of HCV infection that also include actions in regard of PWID. They also reported on having national guidelines for treatment of HCV infection and separate guidelines for treatment of HCV-infected PWID. In all of these countries, apart from Denmark, there have been several studies on HCV treatment access in PWID. The proportion of treated PWID reached 40% in a French study and 57% in a Greek study [63,64]. In Slovenia, the proportion of PWID treated for HCV increased from 3% to 13% in the first three years after the 2007 introduction of the national strategy, action plan and clinical guidelines; 72% of all the newly treated patients in the period 2008-2010 reported a history of drug use [64]. As for Scotland, the clinical services that were developed with the implementation of the Hepatitis C Action Plan led to a doubling in the number of those who initiated therapy in the years 2010–2011 compared to the period before the 2006 introduction of the plan. Eighty percent of people initiating HCV treatment in 2010–2011 had a history of injecting drugs [65]. These observations support the premise that the national policy context is a crucial factor in determining how much PWID will be able to benefit from HCV treatment interventions. To systematically address the barriers to treatment, several steps could be undertaken, with three essential steps being the establishment of a national strategy, action plan and clinical guidelines on HCV treatment.

Although ever/current drug injectors have treatment efficacy and safety outcomes that are comparable to those for people with no history of drug use [15,66-70], and successful treatment could reduce the likelihood of HCV transmission among PWID by eliminating potential sources of infection [9], international and national treatment guidelines historically have excluded PWID, particularly current injectors, from being treated for hepatitis C [15,70,71]. In 2011, however, the Clinical Practice Guidelines of the European Association for the Study of the Liver (EASL) were revised to endorse the provision of HCV treatment to certain PWID based on an individual approach [72]. The 2014 update to these guidelines goes even further in terms of considering substance users as a treatable patient group at risk provided they wish to receive treatment and are willing and able to maintain follow-up visits [62].

It is well recognised that the extremely varied political, economic and social circumstances of different European countries lead to very different health needs and health outcomes at the national level [73]. Furthermore, HCV infection shows great diversity among European countries, not only in its prevalence in the general population and among PWID [8,12], but also in the treatment of hepatitis C. Treatment funding, availability of anti-HCV drugs and the settings for HCV treatment in PWID vary immensely among the European countries. Policies, strategies and guidelines for HCV management therefore must be tailored to the specific national or sub-national context [73].

Several studies have shown that compared to other well-accepted medical interventions, HCV treatment is cost-effective [7]. According to the 2013 Global Policy Report on the Prevention and Control of Viral Hepatitis in WHO Member States, publicly funded treatment for HCV is available in 34 out of 44 Member States from the European Region that responded [73]. Among the participating Member States that were also included in our study, all, except Switzerland, have publicly funded HCV treatment; the data for Albania and Slovakia were not available. Norway, Portugal, Romania, Greece and Scotland, which were included in our study, did not participate in the WHO survey. Among 25 countries of our survey that also provided WHO data on funding, 14 of them reported to have treatment guidelines that include PWID, whereas only eight countries reported on having a national strategy.

Interestingly, in Latvia, which reported having a national strategy and treatment guidelines that include PWID, only 75% of the total treatment cost is publicly funded [73]. In Finland, which according to our survey is in the process of developing a national strategy and action plan for HCV treatment, active injecting drug use is an absolute contraindication for publicly funded HCV treatment [73]. According to the results of our study, only 27% of the respondents reported having separate guidelines for HCV treatment in active drug users; however, this treatment is publicly funded [73]. Switzerland provides no public funding as well as no national strategy and guidelines. As for Scotland, which is regarded globally as a model of good practice, the government made a serious financial investment to support the 2006 launch and subsequent implementation of the Hepatitis C Action Plan, with a special focus on PWID [74].

According to the 2013 WHO Global Policy Report, only 38.6% of Member States in the European region reported having boceprevir and telaprevir on national essential medicine lists or subsidised by governments [73]. It is of special concern that even pegylated interferon was reported not to be present in 20.5% of countries, or ribavirin in 13.6% of countries. In the last year, HCV treatment success and safety have advanced tremendously thanks to new treatment options. Unfortunately, the extremely high prices of the new direct acting antivirals (DDAs) will limit their availability in many European countries [75,76]. Therefore, national strategies and action plans might turn out to be crucial in promoting access to the new drugs. However, it is worth mentioning that no studies of DAAs have been performed in PWID populations thus far. Even the studies of boceprevir and telaprevir have excluded PWID other than OST patients, which means that no efficacy and safety data exist for this population [62].

Beside the treatment of HCV, medical health interventions for PWID also include various programmes for the management of drug addiction, including OST, which has been shown to facilitate HCV treatment and reduce HCV acquisition among PWID [15]. It is of special concern that some European countries are reluctant to use OST [77]. However, almost half of the countries involved in this study reported on having separate guidelines for HCV treatment in PWID on OST. Already existing settings for the management of drug use and for the treatment of HCV infection in PWID vary immensely among the European countries. Therefore, various models have been proposed for HCV treatment in PWID to be performed inter-disciplinarily in either community-based clinics, drug treatment settings, viral hepatitis clinics/hospitals or being integrated into the primary/secondary/tertiary medical setting [78]. The EASL Clinical Practice Guidelines also call for the treatment of HCV in PWID to be undertaken using a multidisciplinary approach [62]. Countries should decide on the most appropriate model for their local facilities. Slovenia provides an example of a multidisciplinary model of good practice that was introduced through a national strategy, action plan and clinical guidelines. The Slovenian model called for the integration of 18 existing drug treatment centres and five viral hepatitis centres into a national healthcare network for HCV treatment in PWID [50].

There are some limitations to our study. The informants who participated in the survey were selected from the database of the Correlation Network’s Hepatitis C Initiative, and all of them were deeply involved in the field of PWID/HCV infection, most as representatives of non-governmental organizations. In all but two countries, only one person was approached to participate in the survey. The validity of the data was not checked separately. The data capture method of the study allows a potential bias since the organizations with strong motivations, but not necessarily linked to government policy input, can have a differing view and/or experiences to what is a current official policy. Therefore, an exploration of civil society versus political leaders/government replies from European countries on the existence of national strategies, action plans and clinical guidelines for HCV treatment in the general population and PWID in particular might be a matter for further research.

## Conclusions

To the best of our knowledge this is the first study to present data on the existence of national strategies, action plans and guidelines on HCV treatment in 33 European countries, and to present data of this nature relating specifically to PWID.

The results of the survey provide data on how European countries address the problem of HCV treatment in PWID and try to achieve hepatitis C policy objectives with regards to treatment. The survey results highlight major gaps that should be addressed in order to improve hepatitis C treatment at the national level, particularly among PWID. Given the burden of HCV-related morbidity and mortality in this at-risk population, the management of HCV infection in PWID should become a healthcare priority in all European countries. Our data demonstrate some awareness of the threat that HCV poses to PWID in Europe and indicate that decision-makers in some countries are willing to consider this subgroup of HCV patients for treatment. With several new direct acting antivirals on the horizon, there is hope that HCV infection can be cured in the majority of patients in the nearest future. However, the most highly potent regimens may remain under-utilised until the group at greatest risk for HCV infection is recognized as urgently needing access to this treatment.

## Competing interests

The authors declare that they have no competing interests regarding this manuscript.

## Authors' contributions

MM carried out the conceptualisation, design, and coordination of the study, drafted the manuscript and prepared the final manuscript. JVZ and SG performed the study analysis and assisted with drafting the manuscript. ES prepared the database of respondents and carried out the collection of data. JVL provided considerable input during the drafting and finalization of the manuscript. All authors read and approved the final manuscript.

## Declarations

This article has been published as part of *BMC Infectious Diseases* Volume 14 Supplement 6, 2014: Viral Hepatitis in Europe. The full contents of the supplement are available online at http://www.biomedcentral.com/bmcinfectdis/supplements/14/S6. The publication charges for this supplement were funded by AbbVie as an unrestricted grant to Rigshospitalet, the University of Copenhagen. AbbVie further funded the printing of the supplement with additional financial support from the Drug Prevention and Information Programme (DPIP) of the European Union.
